# Laparoscopic versus Open Radical Cystectomy in Bladder Cancer: A Systematic Review and Meta-Analysis of Comparative Studies

**DOI:** 10.1371/journal.pone.0095667

**Published:** 2014-05-16

**Authors:** Kun Tang, Heng Li, Ding Xia, Zhiquan Hu, Qianyuan Zhuang, Jihong Liu, Hua Xu, Zhangqun Ye

**Affiliations:** Department of Urology, Tongji Hospital, Tongji Medical College, Huazhong University of Science and Technology, Wuhan, China; Eberhard-Karls University, Germany

## Abstract

**Background and Objective:**

More recently laparoscopic radical cystectomy (LRC) has increasingly been an attractive alternative to open radical cystectomy (ORC) and many centers have reported their early experiences in the treatment of bladder cancer. Evaluate the safety and efficacy of LRC compared with ORC in the treatment of bladder cancer.

**Methods:**

A systematic search of Medline, Scopus, and the Cochrane Library was performed up to Mar 1, 2013. Outcomes of interest assessing the two techniques included demographic and clinical baseline characteristics, perioperative, pathologic and oncological variables, and post-op neobladder function and complications.

**Results:**

Sixteen eligible trials evaluating LRC vs ORC were identified including seven prospective and nine retrospective studies. Although LRC was associated with longer operative time (*p*<0.001), patients might benefit from significantly fewer overall complications (*p*<0.001), less blood loss (*p*<0.001), shorter length of hospital stay (*p*<0.001), less need of blood transfusion (*p*<0.001), less narcotic analgesic requirement (*p*<0.001), shorter time to ambulation (*p* = 0.03), shorter time to regular diet (*p*<0.001), fewer positive surgical margins (*p* = 0.006), fewer positive lymph node (*p* = 0.05), lower distant metastasis rate (*p* = 0.05) and fewer death (*p* = 0.004). There was no significant difference in other demographic parameters except for a lower ASA score (*p* = 0.01) in LRC while post-op neobladder function were similar between the two groups.

**Conclusions:**

Our data suggest that LRC appears to be a safe, feasible and minimally invasive alternative to ORC with reliable perioperative safety, pathologic & oncologic efficacy, comparable post-op neobladder function and fewer complications. Because of the inherent limitations of the included studies, further large sample prospective, multi-centric, long-term follow-up studies and randomized control trials should be undertaken to confirm our findings.

## Introduction

Bladder cancer (BCa) is the fourth and fifth most commonly diagnosed malignancy in the United States and Europe respectively [1]. Open radical cystectomy (ORC) remains the gold standard of care for patients with muscle-invasive organ-confined bladder cancer, providing efficacy with regard to local control and long-term disease-free survival [2]–[12]. Despite a better understanding of pelvic anatomy and improved surgical techniques, ORC is still associated with significant perioperative complications in which intraoperative blood loss is one of the inevitable events even when performed by experienced surgeons [5], [7], [13]–[17]. In an attempt to minimize intraoperative blood loss and decrease perioperative complications of ORC, the most notable change is the increased application of minimally invasive laparoscopic surgery in the management of bladder cancer.

The first case of laparoscopic cystectomy was reported in 1992 by Parra et al. [18], when they performed simple cystectomy for a 27-year-old woman with post-tramatic paraplegia complicated with benign pyocystis and retained bladder after urinary diversion. In recent years, a number of investigators have begun to report case series of minimally invasive laparoscopic approach of radical cystectomy compared with ORC, which demonstrated the surgical feasibility with the potential of fewer complications, decreased pain, shorter hospital stay and decreased intraoperative blood loss while offering the same functional and oncologic results [19]–[34]. Thus, we conducted a systematic review and meta-analysis of the literatures on the feasibility and advantage of LRC versus ORC in terms of demographic and clinical baseline characteristics, perioperative, pathologic and oncological variables, post-op neobladder function and complications.

## Methods

### Study Selection

A systematic search of Medline, Scopus, and the Cochrane Library was performed to identify all studies published up to Mar 1, 2013 which compared LRC with ORC with following MESH search headings: “comparative studies”, “laparoscopic”, “open”, “cystectomy” and “bladder cancer”. The “related articles” function was used to broaden the search, and all abstracts, studies, and citations were reviewed.

### Inclusion Criteria and Exclusion Criteria

To be included in the analysis, studies were required to: (i) the comparison LRC with ORC, (ii) report on at least one outcome of interest mentioned below, (iii) clearly document the techniques as laparoscopic cystectomy, and (iv) clearly document indications for cystectomy with bladder cancer.

Studies were excluded in the meta-analysis if: (i) the inclusion criteria were not met, (ii) no outcomes of interest (specified later) were reported or impossible to calculate or extrapolate the necessary data for either LRC or ORC from the published results, (iii) studies focusing on pure laparoscopic procedures single-site and/or on robotic assisted techniques, and (iv) children were included in the studies.

### Data Extraction and Outcomes of Interest

Two reviewers (TK and XH) independently extracted the following data including: first author, year of publication, country, study interval, study design, indications for operation, number of patients who underwent LRC or ORC, rate of conversion from laparoscopic to open technique, characteristics of the study population, and outcomes of interest. All disagreements about eligibility were resolved by a third reviewer (ZX) by discussion until a consensus was reached. In all cases of missing or incomplete data, the corresponding authors were contacted, but no additional information was provided.

The following outcomes were extracted to compare LRC and ORC.

Demographic and clinical baseline characteristics. Demographic variables were a series of patients’ baseline characteristics including: age, proportion of males, BMI, ASA score, previous surgery history, clinical stage and diversion type (ileal conduit & ileal neobladder).

Perioperative variables. Perioperative variables included operating time, estimated blood loss (EBL), length of hospital stay (LOS), blood transfusion rate, time to ambulation, time to regular diet and narcotic analgesic requirement.

Pathologic and oncological variables included postoperative pathologic stage (pT0,Ta,Tis,T1, pT2, pT3, pT4), pathologic grade (grade1,2,3), positive surgical margins, mean lymph node yield, positive lymph node, local recurrence, distant metastasis, cancer-free survival and death.

Post-op neobladder function assessment variables included maximum urinary flow rate (Qmax), neobladder capacity, intravesical pressure (IVP), residual urine volume (RUV), day time continence rate and night time continence rate.

Postoperative complications variables were overall complications, major and minor complications, and a series of comprehensive and meticulous variables of all complications including infectious disease (wound infection, pulmonary infection, urinary tract infection [UTI], gastrointestinal [GI] infection and systemic sepsis), wound dehiscence, neurologic, renal fistula/leak, ureteric obstruction, GI fistula/leak, ileus and thromboembolic (deep vein thrombosis/pulmonary embolus [DVT/PE]).

### Study Quality and Level of Evidence

The level of evidence (LOE) of included studies was rated according to criteria by the Centre for Evidence-Based Medicine in Oxford, UK [35]. Studies achieved a score of 3b or higher levels indicated to be a higher quality. Two reviewers (K.T. and H. X.) independently assessed the quality of the studies and disagreement was resolved by consensus.

## Statistical Analysis

The present meta-analysis was performed according to the recommendations of the Cochrane Collaboration and the Quality of Reporting of Meta-analyses (QUORUM) guidelines [36]. The weighted mean differences (WMDs) and the odds ratios (ORs) were used to compare continuous and dichotomous variables, respectively. If continuous variables were measured in different units, the standardized mean differences (SMDs) were used. All outcomes were reported with 95% CIs (95% confidence interval). For continuous variables (eg, operating time and length of hospital stay), we calculated the difference in mean values and the 95% CI between LRC and ORC. This method requires that the study report the standard errors of the mean, the standard deviations, or the CIs. However, some studies that did not report any of these parameters but presented continuous data as medians and ranges, under this circumstance we made an approximate transformation using the technique described by Hozo [37]. For dichotomous variables derived from contingency tables (eg, complication rate), the ORs and 95% CI were computed. An OR significantly <1 favores LRC, whereas an OR significantly >1 favores ORC. All *p* values are two-tailed with a significant level at 0.05.

A fixed-effects (FE) meta-analysis was performed, and the quantity of heterogeneity was assessed using *χ^2^* and *I^2^* statistics with significance set at *p*<0.10 providing evidence of significant heterogeneity, For outcomes detected with higher values of *I^2^* and the *χ^2^* statistic signified increasing levels of inconsistency between studies and significant interstudy heterogeneity, then a random-effects (RE) meta- analysis model was adopted. Egger’s test was used and funnel plots were exploited to determine the presence of publication bias.

Sensitivity analysis was carried out in high-quality studies which achieved a score ≥3b as referred before. Variables were pooled only if outcomes reported by three or more studies in the overall meta-analysis.

Statistical analysis was performed using Review Manager (RevMan) Version 5.1 (The Cochrane Collaboration, Oxford, London, UK) and the metareg procedure STATA 12.0 (Stata Corp, College Station, TX).

## Meta-Analysis Results

### Characteristics of Eligible Studies

**Figure 1:**
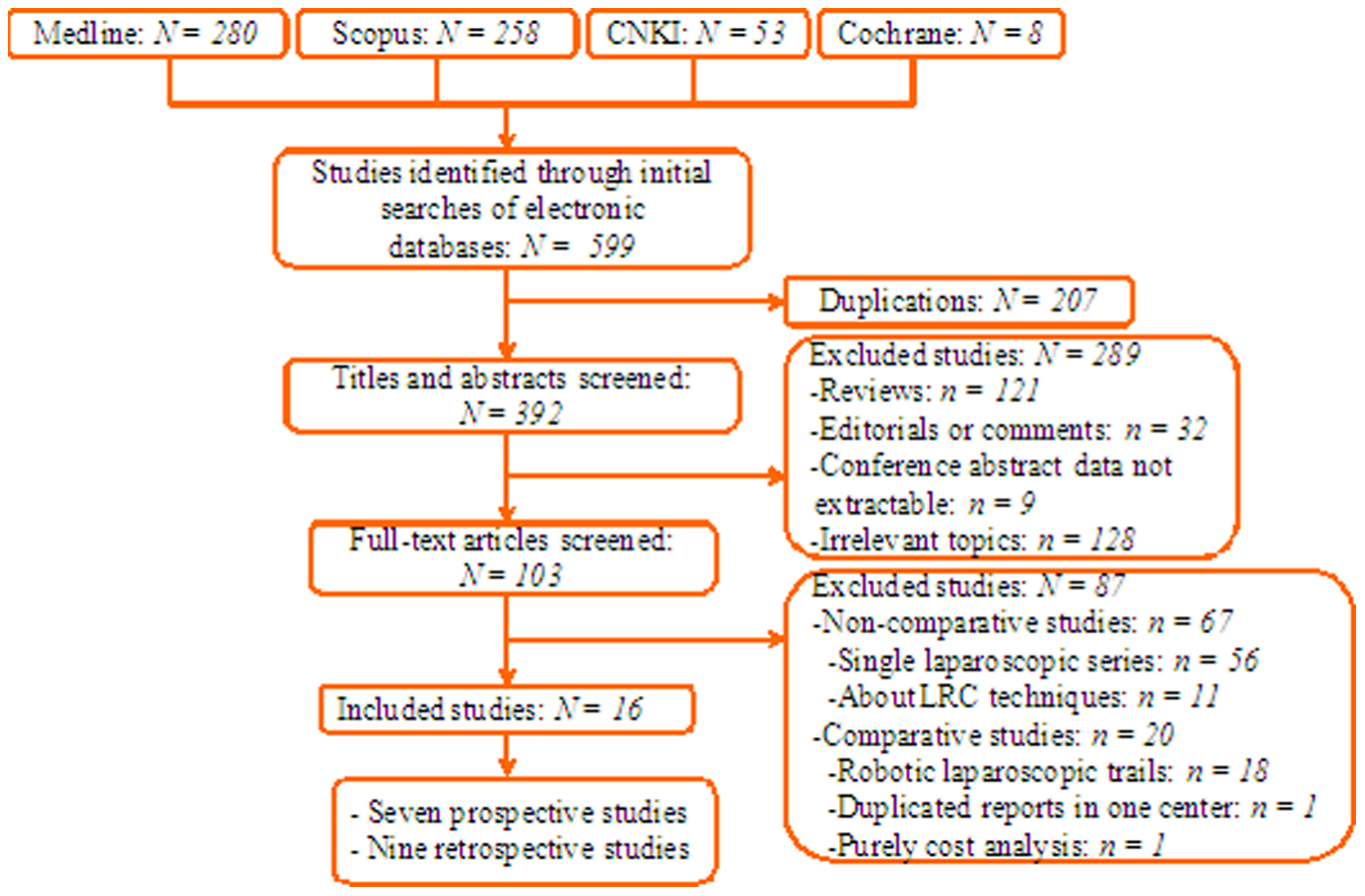
Flow chart of studies identified, included, and excluded.

Sixteen trials including 1165 cases (545 cases and 620 controls) assessing LRC vs. ORC fulfilled the predefined inclusion criteria and were considered suitable for meta-analysis including seven prospective and nine retrospective studies (Fig. 1).

### Quality of the Studies and Level of Evidence

We utilized the US Preventive Services Task Force grading system [35] to assess the quality of every study included in our meta-analysis. Above the seven prospective and nine retrospective studies, eleven studies’ [22], [23], [25], [27], [29], [31] LOE scored ≥3b and were considered to be of high quality. Also, the demographic, rate of conversion to open, matching variables of LRC vs ORC and follow-up time were extracted individually from each study and listed Table 1.

**Table 1:** Characteristics of included studies.

First author, year	Country	Study interval	Design	LOE‡	No. of patients: LRC/ORC	Conversion, N(%)	Matching/Comparable*	Follow-up, mo†:LRC/ORCA
Akin, 2013 [19]	Turkey	2008–2011	Prospective	3b	15/15	0	1,2,3,5,6,8	40/35
Basillote, 2004 [20]	USA	2001–2003	Retrospective	3b	13/11.	2/13(15.4%)	1,2,3,4,6,7,8	NA
Ha, 2010 [21]	Korea	2003–2008	Retrospective	3b	36/34	0	1,2,3,6	22(3–58)/67(6–128)
Haber, 2008 [22]	USA	NA	Retrospective	4	50/50	NA	1,2,3,6	NA
Hemal, 2007 [23]	India	1999–2005	Prospective	3b	30/35	0	1,2,3,4,6	NA
Huang, 2005 [25]	China	1994–2004	Retrospective	4	33/48	0	1,2,6	13(1–21)/24(12–96)
Huang, 2008 [24]	China	1994–2007	Retrospective	4	108/63	0	1,2,3,4,5,6	(12–65)/(18–168)
Jin, 2012 [26]	China	2007–2012	Retrospective	4	32/35	0	1,2,6,7	(6–76)
Julien, 2009 [27]	France	2003–2007	Prospective	3b	38/30	2/38(5.3%)	1,2,3,4,5,6,8	NA
Khan, 2012 [28]	UK	2003–2008	Prospective	3b	58/52	0	1,2,6,8	39.4
Porpiglia, 2007 [30]	Italy	2002–2005	Prospective	3b	20/22	1/20(5%)	1,2,4,6,7,8	19.5(3–37)/19.2(3–39)
Taylor, 2004 [31]	USA	2002–2003	Prospective	3b	8./8	0	1,2,3,4,6,8	NA
Wang^1^, 2010 [32]	China	2006–2008	Prospective	3b	14/24	NA	1,2,3,4,6,8	NA
Wang^2^, 2010 [33]	China	2004–2007	Retrospective	4	31/39	0	1,2,3,5,6,8	18(1–34)/20(1–36)
Wu, 2009 [29]	China	2006–2009	Retrospective	3b	15/23	0	1,2,6,7,8	(3–8)/(3–24)
Zhang, 2010 [34]	China	2007–2009	Retrospective	3b	35/36	2/35(5.7%)	1,2,4,5,6,8	51.4 (1–159)

LRC =  laparoscopic radical cystectomy; ORC =  open radical cystectomy; LOE = Level of evidence; NA = data not available.

*Matching/comparable variables: 1 =  age, 2 =  gender, 3 =  BMI, 4 =  ASA, 5 =  Previous surgery history, 6 =  indication, 7 =  clinical stage, 8 =  diversion type;

† Mean±SD or Median(Range);

‡ Based on US Preventive Services Task Force grading system.

### Outcomes of Demographic and Clinical Characteristics

LRC seemed to have a lower ASA score (WMD: −0.09; 95% CI, −0.15 to −0.02; *p* = 0.01) (Table 2). There was no significant difference with respect to age, proportion of males, BMI, history of previous surgery, organ-confined ≤cT2, non-organ confined ≥cT3, ileal conduit and ileal neobladder.

**Table 2:** Overall analysis of demographic and clinical characteristics comparing LRC and ORC.

Outcome of interest	No. of studies	No. of patients, LRC/ORC	OR/WMD (95% CI)	*p*-value	Study heterogeneity				Egger’s test (*p* value)
					Chi^2^	df	*I^2^*	*p*-value	
Age (years)	16	534/522	4.08 [3.58, 4.57]†	0.23	92.67	15	84%	**<0.001**	0.69
Proportion of males	14	506/496	1.41 [0.96, 2.09]	0.08	14.71	13	12%	0.33	0.74
BMI (kg/m^2^)	10	343/309	0.31 [−0.58, 1.21]†	0.49	36.30	9	75%	**<0.001**	0.79
ASA score	8	264/229	−0.09 [−0.15, −0.02]†	**0.01**	9.93	7	29%	0.19	**0.03**
Previous surgery	5	227/183	0.58 [0.23, 1.46]	0.25	11.94	4	66%	0.02	0.28
Clinical stage									
Organ confined ≤cT2	3	45/54	1.26 [0.41, 3.89]	0.69	1.91	2	0%	0.38	0.91
Non-organ confined ≥cT3	3	45/54	0.80 [0.26, 2.47]	0.69	1.91	2	0%	0.38	0.16
Diversion type									
Ileal conduit	5	176/181	0.60 [0.35, 1.01]	0.06	6.90	4	42%	0.14	0.17
Ileal neobladder	5	176/181	1.68 [0.99, 2.85]	0.06	7.09	4	44%	0.13	0.60

CI = Confidence interval; OR = odds ratio; WMD = weighted mean difference; LRC =  laparoscopic radical cystectomy; ORC =  open radical cystectomy; ASA = American Society of Anesthesiologists score.

† Values of WMD;

*Statistically significant results are shown in bold.

### Outcomes of Perioperative Variables

**Figure 2:**
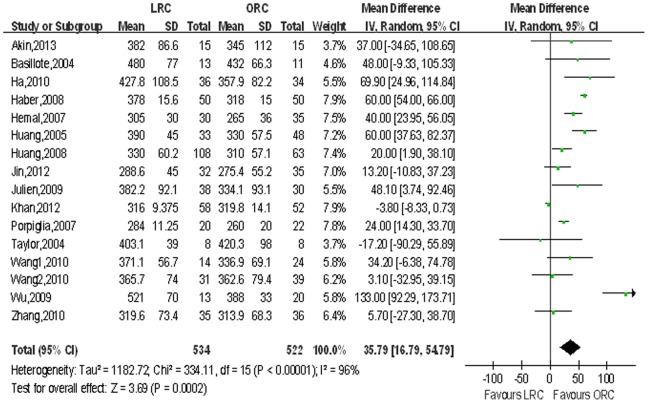
Forest plot and meta-analysis of operating time. LRC = laparoscopic radical cystectomy; ORC = open radical cystectomy.

Operating time and narcotic analgesic requirement. Pooled data from the 16 studies [20]–[24], [26]–[32] that reported operating time and 6 for narcotic analgesic requirement between LRC and ORC, and LRC was associated with longer operative time (WMD 35.79 min; 95% CI, 16.79–54.79; p<0.001; Table 3) and less need of narcotic analgesic (WMD: −29.55 mg; 95% CI, −43.70 to −15.39; p<0.001; Table 3). Fig. 2,3 show forest plots for operating time and narcotic analgesic requirement.

**Figure 3:**
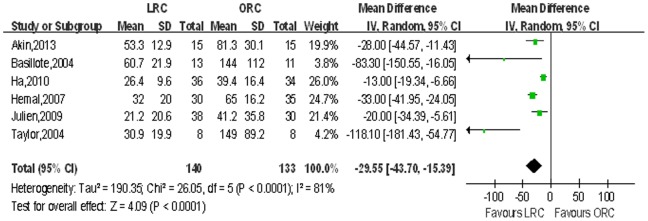
Forest plot and meta-analysis of narcotic analgesic requirement. LRC = laparoscopic radical cystectomy; ORC = open radical cystectomy.

**Table 3:** Overall analysis of perioperative outcomes comparing LRC and ORC.

Outcome of interest	No. of studies	No. of patients, LRC/ORC	WMD/OR (95% CI)	*p*-value	Study heterogeneity				Egger’s test (*p* value)
					Chi^2^	df	*I^2^*	*p*-value	
Operating time, min	16	534/522	35.79 [16.79, 54.79]	**<0.001**	334.11	15	96%	**<0.001**	0.08
EBL, mL	16	534/522	−467.32 [−636.72, −297.91]	**<0.001**	465.70	15	97%	**<0.001**	0.11
LOS, days	16	534/522	−2.72 [−3.63, −1.80]	**<0.001**	38.55	15	61%	**<0.001**	0.35
Blood transfusion rate	9	284/281	0.20 [0.14, 0.29]†	**<0.001**	12.24	8	36%	0.13	0.64
Time to ambulation, days	3	118/119	−1.69 [−3.24, −0.14]	**0.03**	13.22	2	85%	**0.001**	0.14
Time to regular diet, days	15	476/470	−1.53 [−1.95, −1.11]	**<0.001**	256.44	14	95%	**<0.001**	**0.01**
Narcotic analgesic requirement, mg	6	140/133	−29.55 [−43.70, −15.39]	**<0.001**	26.05	5	81%	**<0.001**	0.37

CI = Confidence interval; OR = odds ratio; WMD = weighted mean difference; LRC =  laproscopic radical cystectomy; ORC =  open radical cystectomy; EBL = estimated blood loss; LOS = length of hospital stay.

† Values of OR;

*Statistically significant results are shown in bold.

**Figure 4:**
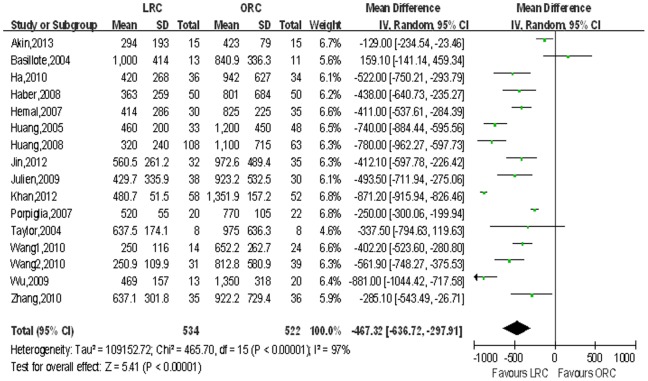
Forest plot and meta-analysis of estimated blood loss (EBL). LRC = laparoscopic radical cystectomy; ORC = open radical cystectomy.

Estimated blood loss (EBL) and blood transfusion rate. We extracted estimated blood loss from 16 studies [20]–[23], [28]–[31] and blood transfusion rate from 9 studies [26], [28], [32]. There were statistically significant less blood loss (WMD: −467.32 ml; 95% CI, −636.72 to −297.91; p<0.001; Table 3) and lower blood transfusion rate (OR: 0.13; 95% CI, 0.03–0.46; p = 0.002; Table 3) in the LRC group compared with ORC group. Fig. 4,5 show forest plots for EBL and blood transfusion rate respectively.

**Figure 5:**
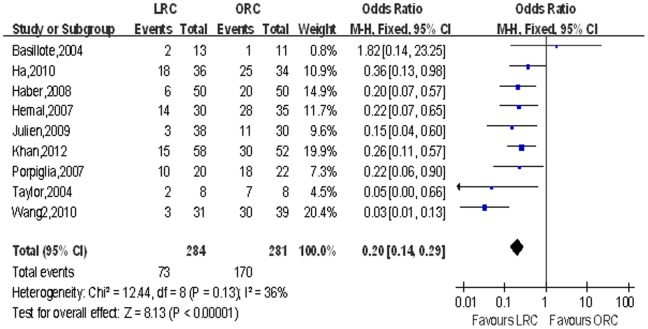
Forest plot and meta-analysis of blood transfusion rate. LRC = laparoscopic radical cystectomy; ORC = open radical cystectomy.

### Postoperative Recovery

**Figure 6:**
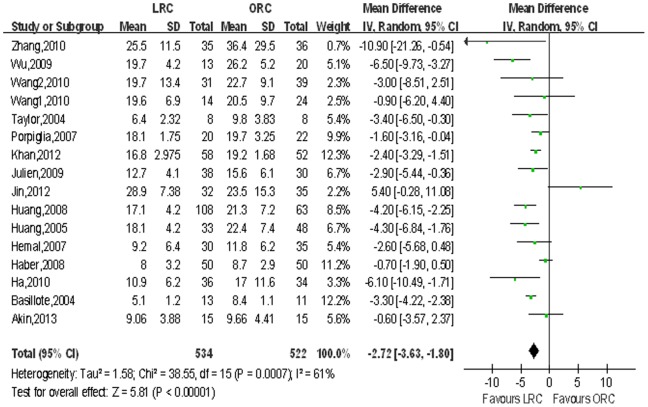
Forest plot and meta-analysis of length of hospital stay (LOS). LRC = laparoscopic radical cystectomy; ORC = open radical cystectomy.

**Figure 7:**
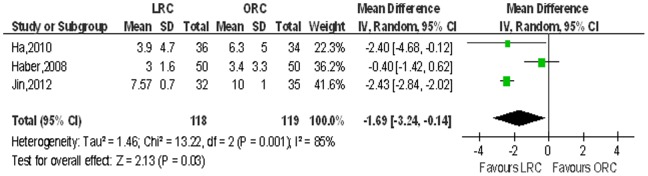
Forest plot and meta-analysis of time to regular diet. LRC = laparoscopic radical cystectomy; ORC = open radical cystectomy.

16 studies [20]–[24], [26]–[32] including 1056 patients on length of hospital stay (LOS), 3 studies [21], [23], [27], [32] on time to ambulation and 15 studies [21], [23], [27], [32] on time to regular diet were reported respectively, and the pooled data showed a significant difference favoring the LRC group associated with shortened hospital stay (WMD: −2.72 d; 95% CI, −3.63 to −1.80; *p*<0.001; Table 3), shorter time to ambulation (WMD: −1.69 d; 95% CI, −3.24 to −0.14; *p* = 0.03; Table 3) and regular diet (WMD: −1.53 d; 95% CI, −1.59 to −1.11; *p*<0.001; Table 3). Fig. 6,7,8 show the forest plots for LOS, time to ambulation and time to regular diet respectively.

**Figure 8:**
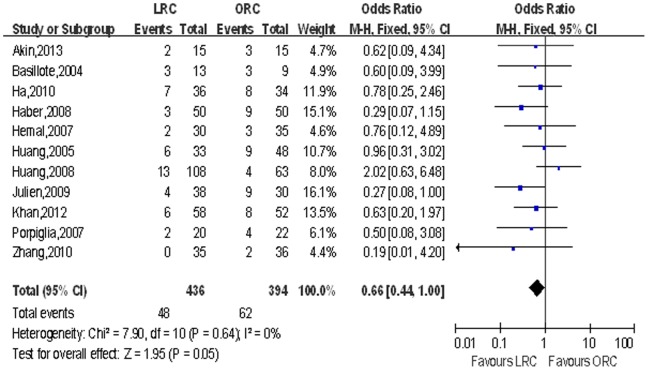
Forest plot and meta-analysis of time to regular diet. LRC = laparoscopic radical cystectomy; ORC = open radical cystectomy.

### Outcomes of Pathologic and Oncological Variables

Pathologic stage and pathologic grade. A significantly higher proportion of organ confined ≤pT2 was observed in LRC compared to ORC (OR: 1.33; 95% CI, 1.01–1.73; *p* = 0.04), but there was no significant difference in other stages or pathologic grades (all *p*>0.05, Table 4).

**Table 4:** Overall analysis of pathologic and oncological outcomes comparing LRC and ORC.

Outcome of interest	No. of studies	No. of patients, LRC/ORC	OR/WMD (95% CI)	*p*-value	Study heterogeneity				Egger’s test (*p* value)
					Chi^2^	df	*I^2^*	*p*-value	
Pathologic stage									
pT0,Ta,Tis,T1	12	413/384	1.27 [0.89, 1.82]	0.19	3.22	11	0%	0.99	0.93
pT2	14	478/467	1.13 [0.86, 1.49]	0.46	8.33	13	0%	0.81	**0.0008**
pT3	13	463/452	0.84 [0.63, 1.14]	0.27	16.52	12	27%	0.17	**0.05**
pT4	8	340/300	0.59 [0.34, 1.03]	0.06	10.89	7	36%	0.14	0.31
Organ confined ≤pT2	14	513/502	1.33 [1.01, 1.73]	**0.04**	12.37	13	0%	0.50	**0.02**
Non-organ confined pT3–T4	14	513/502	0.80 [0.61, 1.06]	0.12	14.92	13	13%	0.31	**0.01**
Pathologic grade									
Grade 1	5	221/193	0.92 [0.48, 1.75]	0.79	1.29	4	0%	0.86	0.37
Grade 2	7	266/243	1.34 [0.92, 1.94]	0.13	8.35	6	28%	0.21	**0.05**
Grade 3	7	266/243	0.78 [0.53, 1.15]	0.21	9.39	6	36%	0.15	0.19
Positive surgical margins	7	334/281	0.35 [0.16, 0.73]	**0.006**	3.31	6	0%	0.77	0.30
Mean lymph node yield, n	8	355/301	0.53 [−1.42, 2.48]†	0.59	83.18	7	92%	**<0.001**	0.95
Positive lymph node	11	436/394	0.66 [0.44, 1.00]	**0.05**	7.90	10	0%	0.64	0.24
Local recurrence	4	207/180	1.21 [0.37, 3.95]	0.75	2.71	3	0%	0.44	0.78
Distant metastasis	6	254/230	0.56 [0.31, 1.01]	**0.05**	3.67	5	0%	0.60	0.85
Cancer-free survival	4	189/147	1.11 [0.66, 1.86]	0.69	1.36	3	0%	0.71	0.81
Death	6	238/218	0.42 [0.23, 0.76]	**0.004**	3.78	5	0%	0.58	0.90

CI, Confidence interval; OR, odds ratio; WMD, weighted mean difference; LRC =  laparoscopic radical cystectomy; ORC =  open radical cystectomy.

† Values of WMD;

*Statistically significant results are shown in bold.

**Figure 9:**
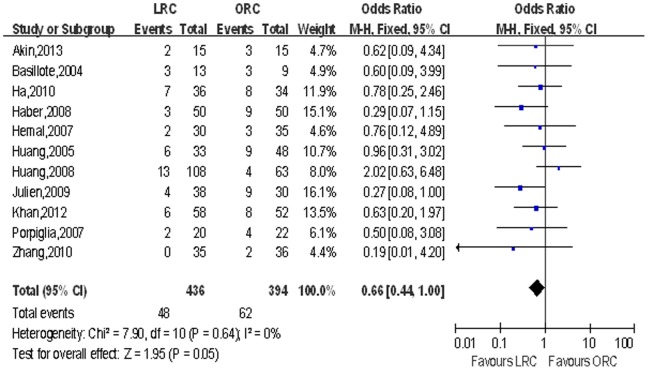
Forest plot and meta-analysis of positive lymph node. LRC = laparoscopic radical cystectomy; ORC = open radical cystectomy.

Mean lymph node yield and positive lymph node. Pooling data of eight studies that counted lymph node yield in 615 patients and 11 studies including 830 patients reported positive lymph node, although there was no significant difference in lymph node yield, LRC group had fewer positive lymph node (OR: 0.66; 95% CI, 0.44–1.00; *p* = 0.05; Table 4; Fig. 9).

**Figure 10:**
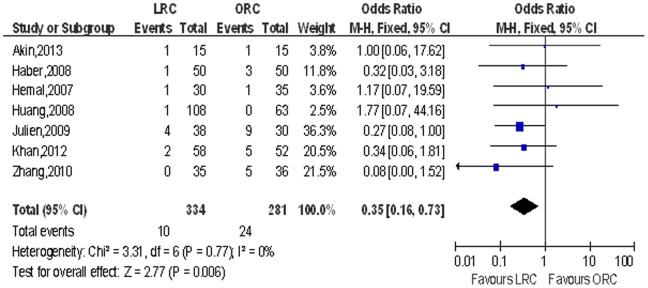
Forest plot and meta-analysis of positive surgical margins. LRC = laparoscopic radical cystectomy; ORC = open radical cystectomy.

Positive surgical margins. Pooling data of seven studies that reported positive surgical margins in 615 patients showed significantly lower PSM rate in LRC than the ORC group (OR: 0.35; 95% CI, 0.16–0.73; *p = *0.006; Table 4; Fig. 10).

**Figure 11:**
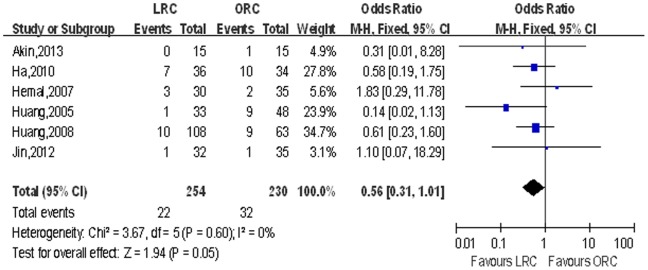
Forest plot and meta-analysis of distant metastasis rate. LRC = laparoscopic radical cystectomy; ORC = open radical cystectomy.

**Figure 12:**
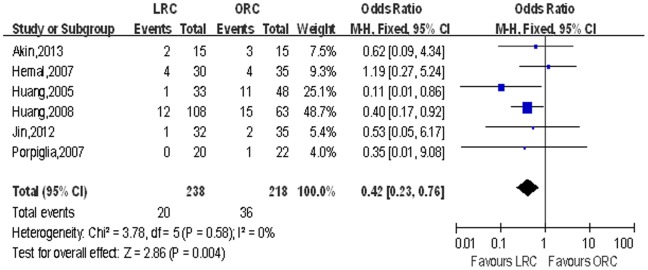
Forest plot and meta-analysis of death. LRC = laparoscopic radical cystectomy; ORC = open radical cystectomy.

Postoperative oncologic recurrence. LRC was associated with lower distant metastasis rate (OR: 0.56; 95% CI, 0.31–1.01; *p* = 0.05; Table 4; Fig. 11) and fewer death (OR: 0.42; 95% CI, 0.23–0.76; *p* = 0.004; Table 4; Fig. 12), and there was no significant difference with regard to local recurrence and cancer-free survival (*p*>0.05).

### Outcomes of Post-op Neobladder Function

Pooling data of four studies including 352 patients that reported post-op neobladder function, revealed no significant difference with regard to Qmax, neobladder capacity, IVP, RUV, day time continence rate or night time continence rate (*p*>0.05; Table 5).

**Table 5:** Overall analysis of post-op neobladder function comparing LRC and ORC.

Outcome of interest	No. of studies	No. of patients,LRC/ORC	WMD/OR (95% CI)	*p*-value	Study heterogeneity				Egger’s test (*p* value)
					Chi^2^	df	*I^2^*	*p*-value	
Q_max_, ml/s	2	46/68	0.89 [−0.75, 2.53]	0.29	3.08	1	68%	0.08	–
Neobladder capacity, ml	4	186/166	3.56 [−4.28, 11.39]	0.37	1.97	3	0%	0.58	0.70
Neobladder IVP, cm H_2_O	3	173/146	0.06 [−1.47, 1.60]	0.94	0.10	2	0%	0.95	0.52
RUV, ml	3	154/131	2.47 [−0.93, 5.87]	0.15	2.94	2	32%	0.23	0.41
Continence rate/day time	3	154/131	1.36 [0.60, 3.09]†	0.46	0.15	2	0%	0.93	0.63
Continence rate/night time	3	154/131	1.09 [0.58, 2.05]†	0.79	0.13	2	0%	0.94	0.70

Q_max_ = Maximum urinary flow rate; IVP = Intravesical pressure; RUV = Residual urine volume.

LRC =  laparoscopic radical cystectomy; ORC =  open radical cystectomy;

† Values of OR.

### Outcomes of Complications

**Figure 13:**
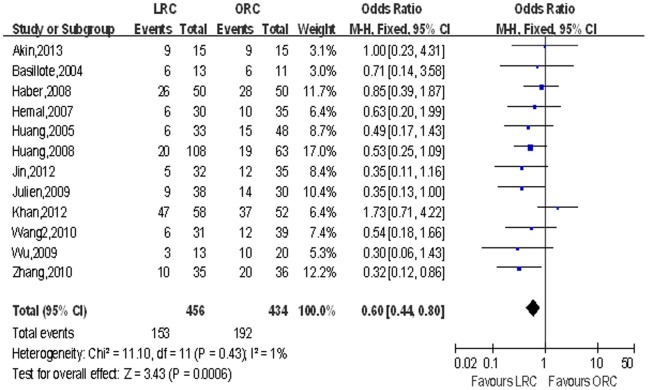
Forest plot and meta-analysis of overall complications. LRC = laparoscopic radical cystectomy; ORC = open radical cystectomy.

Overall complications and major & minor complications. Though there was no significant difference in major complications, pooled data from 12 studies [20]–[23], [26]–[29], [31], [32] including 890 patients reported on complications showed a statistically significant reduction in the overall complications rate in the LRC group compared with the ORC group (OR: 0.60; 95% CI, 0.44–0.80; *p*<0.001; Table 6), especially in minor complications (OR: 0.45; 95% CI, 0.33–0.62; *p*<0.00; Table 6) Fig. 13 shows a forest plot for overall complications.

**Table 6:** Overall analysis of complications comparing LRC and ORC.

Outcome of interest	No. of studies	No. of patients, LRC/ORC	OR (95% CI)	*p*-value	Study heterogeneity				Egger’s test (*p* value)
					Chi^2^	df	*I^2^*	*p*-value	
Overall complications	12	456/434	0.60 [0.44, 0.80]	**<0.001**	11.10	11	1%	0.43	0.50
Major complications	12	456/434	1.04 [0.69, 1.55]	0.86	8.07	11	0%	0.71	0.28
Minor complications	12	456/434	0.45 [0.33, 0.62]	**<0.001**	18.39	11	40%	0.07	0.11
**1. Infectious disease**	11	406/384	0.31 [0.20, 0.49]	**<0.001**	9.80	10	0%	0.46	0.52
Wound infection	8	323/304	0.24 [0.10, 0.57]	**0.001**	3.36	7	0%	0.85	0.23
Pulmonary infection	7	334/299	0.31 [0.14, 0.69]	**0.004**	0.26	6	0%	1.00	0.10
UTI	9	381/348	0.76 [0.40, 1.44]	0.40	5.05	8	0%	0.88	0.92
GI infection	3	88/81	0.35 [0.08, 1.55]	0.17	2.06	2	3%	0.36	0.50
Systemic sepsis	3	115/105	0.15 [0.03, 0.87]	**0.03**	0.40	2	0%	0.82	0.11
**2. Wound Dehiscence**	6	182/188	0.64 [0.21, 1.92]	0.43	2.11	5	0%	0.83	**0.02**
**3. Neurologic**	3	81/76	0.86 [0.24, 3.05]	0.82	2.16	2	7%	0.34	0.06
**4. Renal fistula/leak**	7	291/269	0.63 [0.31, 1.27]	0.19	3.01	6	0%	0.81	0.92
**5. Ureteric obstruction**	4	229/189	1.90 [0.79, 4.54]	0.15	4.89	4	18%	0.30	0.48
**6. GI fistula/leak**	5	246/201	1.17 [0.39, 3.52]	0.78	3.43	4	0%	0.49	0.50
**7. Ileus**	10	399/376	0.54 [0.31, 0.94]	**0.03**	7.37	9	0%	0.60	0.45
**8. Thromboembolic** DVT/PE	5	174/164	0.43 [0.14, 1.35]	0.15	1.78	4	0%	0.75	0.17

CI = Confidence interval; OR, odds ratio; LRC =  laroscopic radical cystectomy; ORC =  open radical cystectomy; UTI = urinary tract infection;

GI = gastrointestinal; DVT =  deep vein thrombosis; PE = pulmonary embolus.

*Statistically significant results are shown in bold.

Comprehensive and meticulous variables of all complications. A comprehensive and meticulous classification of all complications showed that LRC had a statistically significant lower incidence of infectious disease (OR: 0.31; 95% CI, 0.14–0.69; *p*<0.001), wound infection (OR: 0.24; *p* = 0.001), pulmonary infection (OR: 0.31; p = 0.004), systemic sepsis (OR: 0.15; *p* = 0.03) and ileus (OR: 0.54; *p* = 0.03). There were no difference between the LRC and ORC with respect to UTI, GI infection, wound dehiscence, neurologic, renal fistula/leak, ureteric obstruction, GI fistula/leak or thromboembolic (DVT/PE). Heterogeneity between studies was effectively decreased after this comprehensive and meticulous classification of all complications compared with the overall complications.

### Sensitivity Analysis and Publication Bias

Sensitivity analysis (Table 7) was carried out for eleven qualified studies with LOE scored over or equal to 3b. There was no change in the significance of any other outcomes in sensitivity analysis except that distant metastasis (*p = *0.05 vs. *p = *0.48) and death (*p* = 0.004 vs. *p = *0.72) were significantly different compared with the original analysis. Heterogeneity between studies was effectively decreased by the method of sensitivity analysis to a certain extent.

**Table 7:** Sensitivity analysis of eleven high quality studies (LOE≥3b) comparing LRC and ORC.

Outcome of interest	No. of studies	No. of patients, LRC/ORC	OR/WMD (95% CI)	*p*-value	Study heterogeneity			
					Chi^2^	df	*I^2^*	*p*-value
Operating time, min	11	280/287	36.91 [16.41, 57.41]†	**<0.001**	102.09	10	90%	**<0.001**
EBL, mL	11	280/287	−410.00 [−632.28, −187.73]†	**<0.001**	448.82	10	98%	**<0.001**
LOS, days	11	280/287	−2.78 [−3.31, −2.25]†	**<0.001**	16.51	10	39%	0.09
Blood transfusion rate	7	203/192	0.25 [0.16, 0.40]	**<0.001**	4.99	6	0%	0.55
Time to regular diet, days	10	222/235	−1.63 [−2.16, −1.10]†	**<0.001**	35.52	9	75%	**<0.001**
Narcotic analgesic requirement, mg	6	140/133	−29.55 [−43.70, −15.39]†	**<0.001**	26.05	5	81%	**<0.001**
Positive surgical margins	5	176/168	0.31 [0.13, 0.72]	**0.006**	2.35	4	0%	0.67
Positive lymph node	8	245/233	0.54 [0.31, 0.92]	**0.02**	2.13	7	0%	0.95
Distant metastasis	3	81/84	0.73 [0.30, 1.77]	0.48	1.37	2	0%	0.50
Death	3	65/72	0.82 [0.28, 2.44]	0.72	0.59	2	0%	0.74
Overall complications	7	202/199	0.63 [0.41, 0.97]	**0.03**	9.24	6	35%	0.16

CI, Confidence interval; OR, odds ratio; WMD, weighted mean difference; LRC =  laparoscopic radical cystectomy; ORC =  open radical cystectomy.

† Values of WMD;

*Statistically significant results are shown in bold.

The funnel plots and Egger’s tests (Table 2,3,4,5,6) revealed that significant publication bias existed in only seven (ASA score, pT2, pT3, organ confined ≤pT2, non-organ confined pT3–T4, grade 2 and wound dehiscence) of the 54 comparisons performed in the present analysis.

## Discussion

The incidence of bladder cancer rises with age, peaking between age 50 and 70 years, and is three times more common in men than in women which could be verified in all the included studies. As a novel technique emerges, it is natural and appropriate to select favourable patients to try and ensure patients’ safety and optimal outcomes. Many surgeons performing LRC tend to select patients who are generally with a tolerable age, a suitable BMI and a good comorbidity profile early in their series and typically offer the procedure to patients with organ-confined, nonbulky BCa [38]. These favorable selection criteria would also induce those patients a more quick recovery from ORC with a lower probability of transfusion and other complications. Our meta-analysis showed very good baseline characteristics with no significant difference regarding to age, proportion of males, BMI, history of previous surgery, clinical stage or diversion type. Consequently, it is well matched in our included studies; however, the lower ASA score in LRC but with a significant publication bias.

Our meta-analysis demonstrated that patients who underwent LRC had less blood lose and wereless likely to be transfused, which may be partly due to better exposure of surgery fields provided by the minimally invasive laparoscopy. A lower blood transfusion rate may reflect lower complications, with a consequent decrease in blood transfusion needs, shorter time to ambulation and shorter time to regular diet. Moreover, patients received LRC got discharged earlier than those received ORC. Also we evaluated the narcotic analgesic requirement for LRC and ORC, which shows less narcotic analgesic requirement in LRC in consistent with a lower ASA score indicative of less pain, an earlier recovery of bowel function and return to normal activity. Cookson et al speculated that this might be caused by prolonged abdominal retraction and longer incision during ORC [17]. Less postoperative pain and the decreased narcotic analgesic requirement resulted in early recovery of bowel function and ambulation. Considering laparoscopic as a new procedure for cystectomy, it is plausible that ORC might be better in operating time but accumulated experience in LRC may improve this index since the learning curve had already showed a gradual reduction in operating time without compromising the surgical outcomes [39].

Despite significant enthusiasm for LRC in many centers worldwide, there remains concern over pathologic and long-term oncologic results, particularly in patients with more advanced diseases [40]. With regard to the pathologic results, LRC might be associated with a lower stage with more organ confined ≤pT2 since no significant difference is obtained in other stages or pathologic grades. Pelvic lymph-node dissection (PLND) is also an important surgical procedure for RC. Lymphadenectomy not only provides the staging information but is also considered to be curative in patients with nodal metastases. Herr et al. [41] suggested complete PLND with large numbers of lymph nodes yield ensured qualified oncologic outcome. Some authors regarded LN yield as an index of surgical quality with cystectomy [42], and surgeons always concentrated on this main part of the operation and paid more attention to the details as their experience accumulates. Removal of lymph nodes in LRC group was as easyas in the ORC group [43], [44], thus there was no statistical significance in the number of lymph nodes retrieved between LRC and ORC, however, what is interesting was that LRC group had fewer positive lymph node yield which might indicate the patients selected in LRC group were associated with less node metastasis. It is generally believed that qualified RC is indispensable for the treatment of BCa thus oncologic outcomes depend primarily on en bloc dissection of the tumor and perivesical soft tissue and a thorough PLND [45]. Encouragingly, our data showed a significantly lower PSM rate in LRC than that in ORC group, which might result from meticulous dissection due to better perspective of anatomical structure, lower pathological stage and decreased blood loss.

As for the oncologic recurrence, LRC achieved an identical prognosis to ORC in terms of local recurrence and cancer-free survival. We found lower rates of distant metastasis and death in LRC in the original analysis. One possible explanation is that meticulous dissection with lower PSM and fewer positive lymph nodes might give patients the advantage of acquiring better oncologic prognosis in LRC group. However, in the following sensitivity analysis, we observed no significant difference between the two techniques. Therefore, further well-designed large sample long-term follow-up RCTs is required to prove the finding of oncologic outcomes.

The functional outcomes after radical cystectomy neobladder reconstruction largely determine the patients’ post-op quality of life in terms of urinary continence, which is closely related to post-op parameters of Qmax, neobladder capacity, IVP and RUV. There is few literature comparing post-op neobladder function after radical cystectomy between LRC and ORC and only four studies in Chinese were identified, in which three studies adopted orthotopic neobladder reconstruction and one study had ileal conduit. Our included three studies reported daytime continence rates of 91%, 94%, 92% and a nighttime rate of 88%, 82%, 78% respectively, and compared favourably with previously reported values in other single LRC series daytime rate (89–98%) and nighttime rate (78–88%) [46]–[48]. In the present meta-analysis, Qmax, neobladder capacity, IVP, RUV, day time and night time continence rate were similar in the two groups, which demonstrated the efficacy of LRC in post-op neobladder function.

Complications are one of the most important early end points in the evaluation of patient outcome and surgical technique [49]. In this meta-analysis, we attempted to perform a comprehensive and meticulous review of all the common complications after radical cystectomy. To the end, this analysis presented a rigorous comparative series of complications between LRC and ORC. Patients undergoing LRC experienced significantly fewer overall complications, indicating that LRC might be safer and more effective than those undergoing ORC. One possible explanation of the lower complication rate in LRC is less ASA score, lower EBL, less transfusion requirements and minimally invasive surgery. Minor complications identified statistically significant differences, but not significant for major complications. A comprehensive and meticulous classification of all complications showed that LRC had a lower incidence of infectious disease (wound infection, pulmonary infection, systemic sepsis) and ileus. Thus we could draw a conclusion that patients with LRC might be associated with a lower incidience of complications especially in minor complications as infectious diseases and ileus.

To evaluate the impact of study quality on the effect estimation, sensitivity analysis was performed for studies matched for available variables. There was no significant difference in the two sensitivity analysis compared with the original analysis except for distant metastasis and death. The inter-study heterogeneity was not significant for dichotomous outcomes but was significant for most of the continuous variables. Pooling data using the random-effects model might reduce but not abolish the effect of heterogeneity, and it had a downregulation effect in the heterogeneity by the method of sensitivity analysis to some extent.

However, we should admit that there exist certain inherent limitations in the studies included in our meta-analysis which cannot be ignored when interpreting our data. The major limitation of this study is the limited number of well constructed prospective studies. First, there is no RCT included in our analysis. Second, the sample size of some studies included in the analysis is small that the statistical power to detect the difference in the outcomes is limited. Third, the issue of complication grading is one of glitches inherent in the studies included, since it was not always reported in the details of particular complication management. Thus we classified complications into minor and major groups instead of the standard Clavien grading. Additionally, we also grouped the complications into comprehensive and meticulous complications. Although the Clavien classification may be more detailed, this method serves better in this meta-analysis. Moreover, short follow-up time in some patients, marked heterogeneity for several continuous variables, unmeasurable selection bias and the potentially existed significant risk of publication bias may more or less affect the accuracy of the results. Last, there is an inherent problem with reviews on laparoscopic literature in general and in Robotic literature in particular - the technique is company driven in its total extent. Indeed, “the sphere of interest” created by the company is related to that main priority of Robotic laparoscopic techniques.

Nevertheless, our data proved the feasibility and advantage of LRC compared to conventional ORC. Our present meta-analysis comparing LRC and ORC was conducted at an appropriate time with enough data available for extraction. We applied a series of available variables including demographic and clinical baseline characteristics, perioperative, pathologic, oncological variables, post-op neobladder function and complications to compare LRC with ORC and strict criteria to evaluate the quality of the included studies, egger’s test to evaluate the publication bias and the method of sensitivity analysis to minimize the effects of heterogeneity. Here, we provide an up-to-date information worthy of reference on LRC for the treatment of bladder cancer.

## Conclusions

Sixteen trials (545 cases and 620 controls) assessing LRC vs. ORC were considered suitable for meta-analysis including seven prospective and nine retrospective studies. Although LRC was associated with longer operative time, patients with LRC might benefit from significantly fewer overall complications, less blood loss, shorter length of hospital stay, less need of blood transfusion, less narcotic analgesic requirement, shorter time to ambulation, shorter time to regular diet, fewer positive surgical margins, fewer positive lymph node, lower distant metastasis rate and fewer death. Our data suggest that LRC is a safe, feasible and minimally invasive alternative to ORC when performed by experienced surgeons in selected patients. However, despite our rigorous methodological review, because of the inherent limitations of the included studies and the long-term oncologic results are not available, further large sample prospective, multicentric, long-term follow-up studies and Randomized control trials should be undertaken to confirm our findings.

## Supporting Information

Checklist S1 **PRISMA Checklist.**
(DOC)
